# EHR-Based Advanced Care Planning and Late-Stage Cancer Treatment in a Middle-Income Country: A Retrospective Cohort Study

**DOI:** 10.3390/healthcare14020139

**Published:** 2026-01-06

**Authors:** Matheus Hermes Leal, Rafaella Funk, Laura Lima Camargo, Francisca Rego, Rui Nunes

**Affiliations:** 1 Radiotherapy Service, Hospital Ana Nery, Santa Cruz do Sul 96810-002, RS, Brazillaulimacamargo@gmail.com (L.L.C.); 2 Center for Bioethics, Faculty of Medicine, University of Porto, 4200-319 Porto, Portugal; 3 Faculty of Medicine, Universidade de Santa Cruz do Sul (UNISC), Santa Cruz do Sul 96815-900, RS, Brazil

**Keywords:** advanced cancer, end-of-life care, medical futility, late oncologic interventions, advance care planning, palliative care, electronic health records, digital health, Brazil

## Abstract

Background: Cancer-directed treatment near the end of life may represent low-value, high-intensity care and potential medical futility, but data from middle-income countries are limited. This study used digitally documented advanced care planning (ACP) in the electronic health record (EHR) and indicators of late oncologic interventions (LOI) within 15 and 30 days before death to examine end-of-life care in Brazil. Objective: To identify factors associated with LOI near death and to explore whether documented ACP is linked to lower treatment intensity. Design: Retrospective cohort study. Setting/Participants: Adults with metastatic solid tumors who died between January 2022 and December 2023 in two oncology referral hospitals in southern Brazil and had ≥6 months of premortem EHR data. Measurements: LOI were defined as systemic anticancer therapy, radiotherapy, or oncologic surgery within 30 days (LOI-30) or 15 days (LOI-15) before death. Independent predictors were estimated by Poisson regression with robust variance. Results: Among 79 patients, 21.5% received LOI-30 and 8.9% received LOI-15. Breast and lung cancers were the most common primary sites. LOI-30 was independently associated with age < 60 years (relative risk [RR] 3.76; 95% confidence interval [CI] 1.50–9.44), higher education (RR 2.07; 95% CI 1.07–3.99), and lower platelet count (RR 0.96 per 10,000/µL; 95% CI 0.92–0.99). ACP was documented for 19% of patients and was associated with absence of LOI-30. Conclusions: Digitally visible ACP in the EHR was associated with reduced aggressive end-of-life care. Using existing EHR infrastructure to prompt and standardize ACP documentation may help align care with patient values in middle-income countries.

## 1. Introduction

Cancer remains one of the leading causes of death worldwide, with a growing burden in low- and middle-income countries [1]. In advanced disease, many patients receive intensive care in the last weeks of life, raising concerns about medical futility and the quality of end-of-life (EoL) care [2,3].

Indicators such as chemotherapy in the last 14 days of life, repeated emergency department visits, intensive care unit (ICU) admissions and late hospitalizations have been proposed as markers of aggressive EoL care and are now widely used to monitor quality [4,5,6,7,8,9]. Among these indicators, systemic therapy near death has become a benchmark for aggressive care, especially when evidence of benefit is weak or absent [4,5,6,7,8,9,10].

Most evidence on late oncologic interventions (LOI) near death comes from high-income countries with robust palliative care networks and well-developed digital infrastructure [4,5,6,7,8,9,10]. In middle-income settings, patterns of treatment intensity at the end of life may be shaped by different factors, including resource constraints, heterogeneous access to systemic therapy and varying levels of palliative care integration. Brazil, for instance, has a mixed public–private health system in which access to advanced treatments and supportive care is uneven, and local public–private arrangements may shape decisions in distinct ways [8].

Oligometastatic disease, characterized by a limited number of metastatic lesions, has gained attention as a potentially distinct biological state in which aggressive local interventions may prolong survival or improve symptom control [11]. In this context, oncologists may feel justified in offering late disease-directed treatments even in the face of declining performance status and limited time. The conceptual boundary between potentially beneficial oligometastasis-directed therapy and futile escalation near death is, therefore, ethically and clinically delicate.

Advance care planning (ACP)—a structured process that includes discussing prognosis, eliciting patient values and preferences, and documenting goals-of-care—is a key strategy to reduce unwanted aggressive interventions near death and to support patient-centered care [12,13,14,15,16,17,18,19]. ACP has been associated with better alignment between treatments and patient wishes, fewer non-beneficial interventions and improved bereavement outcomes [13,20,21,22]. Yet, in many countries, ACP is underused, especially in oncology and in health systems where palliative care is still consolidating [14,16,17,18,19].

Research on end-of-life (EoL) quality indicators in oncology faces important constraints. EHR-based studies may be limited by heterogeneous documentation practices, incomplete capture of care delivered outside a single institution, and variability in how ACP and goals-of-care discussions are recorded. In middle-income settings, these challenges are often amplified by fragmented care pathways, uneven digital infrastructure, and barriers related to literacy, connectivity, and resource availability.

In the digital era, electronic health records (EHRs) and related tools have the potential to make ACP more visible and actionable. When ACP documentation is easy to locate in the EHR, it can guide clinicians at critical decision points, such as whether to start or continue systemic therapy in the last month of life. Digital indicators—like LOI within 15 or 30 days of death—can be automatically derived from prescription and scheduling systems, allowing services to monitor aggressive EoL care and evaluate quality improvement initiatives. International ACP initiatives already highlight the importance of integrating documentation into digital systems [23,24]. However, empirical evidence on how ACP, LOI and digital features interact in middle-income countries remains limited.

By analyzing structured ACP documentation and digital LOI indicators extracted from the EHR, this study explores how digital visibility may influence treatment decision-making and contribute to reducing potentially futile interventions in a middle-income country context.

In this context, we aimed to investigate, in a Brazilian cohort of patients with metastatic solid tumors, which factors are associated with LOI near death and whether ACP documented in the EHR is linked to a lower likelihood of late treatment. We hypothesized that younger age and higher education would be associated with more LOI, while documented ACP—visible in the digital record—would be associated with a lower likelihood of late treatment.

## 2. Materials and Methods

### 2.1. Study Design and Setting

We conducted a retrospective cohort study in two oncology referral hospitals in southern Brazil: a private non-profit hospital and a philanthropic institution with contracts to the public health system. Both centers provide systemic therapy and radiotherapy to patients from a wide catchment area and use EHRs for most clinical documentation and prescriptions. Reporting followed the Strengthening the Reporting of Observational Studies in Epidemiology (STROBE) guidelines, and the corresponding checklist was used prospectively to structure data collection, analysis and manuscript reporting. A six-month timeframe before death was chosen to capture the typical clinical period during which decisions about further oncologic treatment are made and to balance completeness with the feasibility of chart review.

### 2.2. Participants

We screened all adults (≥18 years) with metastatic solid tumors who died between 1 January 2022 and 31 December 2023 and had at least 6 months of premortem medical records available in the participating oncology services. We excluded patients whose primary diagnosis was haematologic malignancy, those who died suddenly with no previous oncology follow-up and those with incomplete or fragmented records preventing reliable ascertainment of key variables.

### 2.3. Data Sources

We used hospital EHRs as the primary data source, complemented by scanned paper records when needed. The EHR systems included structured fields (e.g., laboratory values, prescriptions, appointment logs) and unstructured clinical notes written by oncologists, radiotherapists, palliative care teams and other professionals. Radiotherapy planning systems and surgical reports were accessed to verify dates and characteristics of cancer-directed interventions. For LOI ascertainment, we prioritized structured EHR sources (medication/prescription module for systemic therapy, radiotherapy scheduling/planning records for radiotherapy start dates, and surgical/procedure logs for oncologic surgery dates). Laboratory values were retrieved from the EHR laboratory interface as structured variables. Unstructured clinical notes were used only to clarify treatment intent or resolve discrepancies between structured sources.

### 2.4. Data Collection and Abstraction

Data were abstracted from electronic and paper medical records using a standardized data collection form developed and piloted by the research team. The form included operational definitions for all variables (demographics, tumor characteristics, comorbidities, ECOG-PS, laboratory values, treatments, ACP documentation and EoL outcomes).

Two investigators with clinical experience independently performed data extraction for all eligible patients. Initially, both extractors reviewed a subset of charts to refine the data collection form and agree on coding rules. Thereafter, each chart was reviewed in detail, and doubtful cases were jointly discussed until consensus. This process was intended to minimize errors and increase the reliability of the dataset.

### 2.5. Variables

Collected variables included:

Demographics: age at death (years), sex and educational level (elementary, high school, higher education).

Clinical characteristics: primary tumor site, comorbidities (including systemic arterial hypertension and diabetes mellitus), history of oligometastatic disease (defined as 1–5 metastatic lesions, in line with the ESTRO/EORTC consensus) [11], and embolic or thrombotic events.

Performance status: ECOG-PS was retrieved from outpatient or inpatient notes 1–2 months before death, whenever available. When more than one value was documented in this period, we selected the value closest to one month before death.

Primary tumor site was categorized as breast, lung, and other solid tumors because of the modest sample size and sparse counts in individual sites. Detailed histology and staging were not consistently available as structured fields across records and were therefore not extracted for analytic modeling.

Laboratory variables: hemoglobin (g/dL), leukocyte count (cells/µL) and platelet count (×10^3^/µL). We searched for results approximately 30–60 days before death, aiming to capture hematologic reserve during the period in which decisions about additional treatment were usually made. When several results were available, we used the value closest to the center of that window.

EoL care and ACP variables: primary treating specialty (clinical oncology, radiotherapy or other), documented ACP or goals-of-care discussions in the EHR (yes/no), formal advance directives (yes/no), palliative care consultation (yes/no), hospitalizations in the last month of life and place of death (hospital vs. out-of-hospital).

### 2.6. Definition of Late Oncologic Interventions (LOI)

Consistent with commonly used indicators of aggressive EoL care [4,5,6,7,8,9], we defined LOI as any of the following cancer-directed treatments delivered near death:

(a) Systemic anticancer therapy: chemotherapy, immunotherapy, targeted therapy or hormone therapy;
(b) Radiotherapy: external beam radiotherapy or brachytherapy with disease-modifying or symptom-directed oncologic intent;
(c) Oncologic surgery: surgical procedures targeting primary or metastatic tumor sites with cytoreductive or disease-modifying intent.

We defined LOI-30 as the occurrence of at least one LOI within 30 days before death, and LOI-15 as at least one LOI within 15 days before death. Non-oncologic procedures such as gastrostomy, nephrostomy, stent placement or other supportive surgeries were not classified as LOI, even when performed in the last weeks of life.

We selected both time windows because the 30-day threshold is widely used in end-of-life quality metrics, whereas the 15-day threshold captures very late interventions close to death; reporting both improves interpretability and acts as a sensitivity analysis across cutoffs.

### 2.7. Outcomes

The primary outcome was LOI-30. LOI-15 was analyzed as a secondary outcome, reflecting very late treatment intensity. Additional descriptive outcomes included the composition of LOI-30 (relative frequency of systemic therapy, radiotherapy and oncologic surgery), the proportion of patients with documented ACP and place of death.

### 2.8. Statistical Analysis

We performed descriptive analyses of sociodemographic and clinical variables using means and standard deviations for continuous variables and frequencies and percentages for categorical variables. Comparisons between patients with and without LOI-30 were initially explored using chi-square or Fisher’s exact tests for categorical variables and *t*-tests or non-parametric equivalents for continuous variables.

To identify independent predictors of LOI-15 and LOI-30, we constructed multivariable Poisson regression models with robust variance and reported adjusted relative risks (RR) with 95% confidence intervals (CI). Potential predictors were selected a priori based on clinical plausibility and prior literature on aggressive EoL care and oligometastatic disease [4,5,6,7,8,9,10,11,25,26,27,28] and included age (<60 vs. ≥60 years), educational level (higher vs. no higher education), presence of comorbidities, systemic arterial hypertension, oligometastatic disease, platelet count (per 10,000/µL increase) and leukocyte count. Because of the modest sample size and limited number of events, we restricted the number of covariates per model to reduce overfitting. Multicollinearity among covariates was assessed using variance-inflation factors (VIF < 2). Sensitivity analyses were performed excluding patients with missing laboratory values; results were consistent. Missing data were handled by complete-case analysis.

All analyses were performed using IBM SPSS Statistics for Windows, version 27.0 (IBM Corp., Armonk, NY, USA). Statistical significance was defined as a two-sided *p*-value < 0.05, and no formal correction for multiple comparisons was applied; analyses should therefore be regarded as exploratory, especially for LOI-15.

### 2.9. Bias

We considered several potential sources of bias. Selection bias could arise because patients with incomplete records or shorter follow-up were excluded; this may have preferentially removed individuals with rapid disease trajectories, who might receive different patterns of EoL care.

Information bias is possible because, as in all retrospective chart reviews, data were dependent on existing documentation. ACP discussions, performance status and comorbidities may have been underreported. Misclassification of LOI is also possible, particularly for procedures at the interface between symptom control and disease modification (e.g., palliative surgery or radiotherapy delivered primarily for symptom relief). Where intent was unclear, we relied on clinical notes and multidisciplinary discussions to classify interventions, but some residual misclassification may remain.

### 2.10. Ethics

The study was approved by the Research Ethics Committee of Universidade de Santa Cruz do Sul (UNISC), in accordance with national regulations and the Declaration of Helsinki. As this was a retrospective review of deceased patients’ records, the requirement for informed consent was waived.

## 3. Results

### 3.1. Patient Characteristics

Seventy-nine patients met the inclusion criteria (Table 1). Overall, patients were older adults with a balanced sex distribution and predominantly elementary or high-school education.

Breast (19.0%) and lung (16.5%) cancers were the most common primary sites, while other tumors comprised the majority (64.6%). Most patients had a high metastatic burden; only 13.9% were oligometastatic (1–5 lesions). Comorbidities were frequent (73.4%), particularly hypertension (60.8%) and diabetes (17.7%). Functional status near death was generally poor (most with ECOG 2–4). ACP/goals-of-care documentation was uncommon (19.0%).

### 3.2. Late Oncologic Interventions and Place of Death

In the last 30 days of life, 17 patients (21.5%) received at least one LOI-30, and 7 (8.9%) received LOI-15 (Table 2). LOI-15/30 were captured from structured EHR prescription/scheduling and procedure logs (as detailed in Methods), whereas ACP/goals-of-care documentation and ECOG-PS relied on EHR documentation plus manual chart review. Among LOI-30 events, systemic anticancer therapy was the most frequent modality (58.8%), followed by radiotherapy (29.4%) and oncologic surgery (11.8%). These rates are broadly comparable to benchmarks from high-income settings, where 10–20% of patients receive chemotherapy in the last 14 days of life and a substantial proportion undergo other aggressive EoL interventions [4,5,6,7,8,9,10,23].

Most LOI occurred in hospital settings and were typically justified in clinical notes as attempts at symptom control (e.g., bleeding, obstruction or pain) or disease stabilization. However, in several cases, ECOG-PS at the time of treatment was ≥2 and laboratory parameters suggested limited physiological reserve, raising questions about the proportionality of further oncologic treatment.

ACP or goals-of-care discussions were documented in 15 patients (19.0%). None of these patients received LOI-30 (0/15), whereas 17 of 64 patients without documented ACP did (26.6%; *p* = 0.032). This corresponds to an absolute risk reduction of 26.6 percentage points and an approximate number needed to treat of about four ACP discussions to prevent one LOI-30, although the small sample size means that confidence intervals are wide and this estimate should be interpreted with caution.

The hospital was the most common place of death. Patients who received LOI-30 were more likely to die in the hospital than those who did not, reflecting more intensive use of acute-care resources near the end of life.

### 3.3. Factors Associated with LOI-15 and LOI-30

Table 3 summarizes the multivariable Poisson regression models for LOI-15 and LOI-30. LOI-15 was associated with higher education (RR 8.74; 95% CI 2.57–29.7; *p* < 0.001) and lower platelet counts (RR 0.94 per 10,000/µL; 95% CI 0.89–0.96; *p* = 0.010). Age < 60 years showed a strong but statistically non-significant trend toward higher risk of LOI-15 (RR 8.19; 95% CI 0.73–91.6; *p* = 0.088), suggesting that younger and more educated patients with limited hematologic reserve were more likely to receive very late oncologic interventions. Confidence intervals for some estimates—particularly for LOI-15—were wide due to the small number of events, and results should therefore be interpreted cautiously as exploratory.

For LOI-30, age < 60 years remained an independent predictor (RR 3.76; 95% CI 1.50–9.44; *p* = 0.005), as did higher education (RR 2.07; 95% CI 1.07–3.99; *p* = 0.030). The presence of any comorbidity (RR 0.69; 95% CI 0.35–1.39; *p* = 0.305), systemic arterial hypertension (RR 0.59; 95% CI 0.28–1.24; *p* = 0.162) and oligometastatic disease (RR 1.38; 95% CI 0.76–2.50; *p* = 0.291) were not significantly associated with LOI-30 in adjusted analysis. Platelet count again showed an inverse association (RR 0.96 per 10,000/µL; 95% CI 0.92–0.99; *p* = 0.020), indicating that patients with lower platelet counts were more likely to receive LOI-30. Leukocyte count was not significantly associated with LOI-30 (RR 0.93 per 1000 cells/mm^3^; 95% CI 0.79–1.08; *p* = 0.337).

## 4. Discussion

In this Brazilian cohort of patients with metastatic solid tumors, approximately one in five received cancer-directed treatment within 30 days of death and almost one in ten received treatment within 15 days. Three findings stand out. First, younger age was consistently associated with LOI near death. Second, a higher educational level was strongly associated with LOI-15 and LOI-30. Third, lower platelet counts were paradoxically associated with a higher likelihood of late treatment. Furthermore, ACP documentation in the EHR, although infrequent, was associated with the absence of LOI-30 in this cohort, suggesting a potential link between digitally visible goals-of-care and reduced late treatment intensity. Taken together, these findings resonate with prior work on aggressive end-of-life (EoL) care in oncology while adding a specific focus on digital records and ACP in a middle-income setting [4,5,6,7,8,9,10,23].

### 4.1. Age, Education, Platelets and Intensity of End-of-Life Care

Younger age was consistently associated with LOI within 30 days of death. This aligns with previous studies showing that younger patients are more likely to receive aggressive EoL care, including chemotherapy and ICU admission near death [4,5,6,7,8,9,23]. Clinicians and families often have greater difficulty accepting the end of disease-directed treatment for younger individuals, which may prolong or escalate therapies even when the anticipated benefit is small and the symptom burden is high. From an ethical standpoint, the tension between hope and realism is particularly pronounced in younger patients, and the risk of potentially futile treatment may be higher if prognostic uncertainty is interpreted as a reason to “try everything” [2,3].

Higher educational level was strongly associated with both LOI-15 and LOI-30. Educational attainment likely reflects a mix of socioeconomic status, health literacy and ability to navigate complex healthcare systems. In mixed public–private systems, more educated patients may have greater access to multiple lines of systemic therapy and to second opinions, which can contribute to prolonged treatment trajectories. At the same time, social privilege may make it easier to mobilize resources in favor of continued treatment, even when the balance between benefit and burden becomes uncertain. Similar gradients in treatment intensity by socioeconomic indicators have been reported in other settings, raising questions about equity and the influence of social capital on EoL decisions [4,6,7,8,9,23].

The association between platelet count and LOI was more unexpected. In both models, lower platelet counts were associated with a higher likelihood of LOI, despite being a marker of reduced hematologic reserve. From a biological perspective, lower platelet counts usually reflect cumulative exposure to systemic therapy, bone marrow involvement or advanced disease. One might, therefore, expect clinicians to be more cautious about additional treatment in patients with low platelet counts. The observed association suggests that, in some cases, therapeutic inertia may prevail: once several treatment lines have been attempted, stopping therapy becomes more difficult than continuing it, even when laboratory parameters point to frailty [10]. This finding should be interpreted as hypothesis-generating and warrants confirmation in larger samples and different contexts.

Oligometastatic disease did not show a statistically significant association with LOI after adjustment. The concept, however, remains ethically and clinically relevant. In some patients with limited metastatic burden, local ablative strategies can offer meaningful survival or symptom benefits [11,24,25,26]. In others, the same interventions near death may have little chance of benefit while carrying substantial burdens. Our data suggest that, at a population level, oligometastatic status alone did not drive LOI, but the fine line between appropriate local control and futile escalation is particularly thin in this group and demands careful, value-sensitive decision-making.

### 4.2. ACP, Digital Records and the Reduction in Potential Futility

The combination of low ACP prevalence (19%) and the complete absence of LOI-30 among patients with documented ACP is particularly relevant to the theme of improving EoL care in the digital era. In our setting, ACP—when it occurred—was recorded in the same EHR used to prescribe chemotherapy, radiotherapy and surgery. When goals-of-care were visible and clearly documented, clinicians did not prescribe LOI-30. This finding is consistent with prior evidence that ACP is associated with less aggressive EoL care, greater concordance between care and patient preferences and better outcomes for families [12,13,14,15,16,17,18,19].

Importantly, these effects were observed without sophisticated digital innovations: a basic EHR, a simple binary variable (“ACP documented: yes/no”) and free-text clinical notes were sufficient to anchor decisions away from potentially futile interventions in the last month of life. This suggests that the main barriers to reducing potentially inappropriate interventions are not technological but cultural and organizational. ACP discussions remain rare and often under-documented, despite the availability of digital infrastructure. Studies across different health systems have shown that ACP uptake depends on clinician training, institutional culture and clear processes, not merely on the legal existence of advance directives or the presence of EHR fields [12,13,14,15,16,17,18,19].

From a digital health perspective, EHRs and related tools can support ACP and EoL decision-making in several ways. International ACP initiatives highlight the importance of integrating documentation into digital systems, using structured fields, flags and shared care plans to ensure that patient preferences are visible across settings [27,28]. In advanced cancer populations, digital tools such as patient portals, telehealth platforms, secure messaging, mobile ACP applications and electronic decision aids may facilitate information sharing, remote conversations and iterative revisiting of preferences over time [19,20,27,28]. For patients and families living far from specialist centers or with limited access to palliative care services, these tools can help overcome geographic barriers and support timely discussions about goals-of-care.

However, the increasing use of digital tools also raises important limitations and risks, particularly in older patients with advanced cancer and in middle-income countries. Digital divides related to age, education, income and connectivity can exacerbate existing inequalities: the very patients who are most vulnerable may have the least access to or comfort with digital platforms. Cognitive impairment, high symptom burden and emotional distress near the end of life may further reduce the ability to engage with digital interfaces. There is also a risk that digitalization may unintentionally depersonalize care if electronic templates and checkboxes are used as substitutes, rather than supports, for deep, empathic conversations between patients, families and clinicians [14,17,19].

These tensions can be fruitfully analyzed through the lens of bioethics. Respect for autonomy requires that digital tools truly help patients understand their prognosis, clarify their values and express preferences in a way that remains meaningful over time, rather than simply collecting signatures or “tick-box” consent [12,13,19]. Beneficence and non-maleficence demand that digital ACP processes reduce suffering and avoid unwanted or disproportionate interventions, rather than adding complexity or generating false reassurance [2,3]. Justice requires attention to equitable access and design: tools must be adapted to different levels of literacy, language and connectivity, so that they do not preferentially benefit only younger, wealthier or more educated patients [17,19,27,28]. Finally, all four principles converge on a central point: digital tools should support, but never replace, the human relationship at the core of palliative and oncology care.

From a practical perspective, our findings suggest that even relatively simple enhancements to existing EHRs could help align care with patient values. Systems could be configured to: (a) prompt ACP discussions when patients cross predefined prognostic thresholds (e.g., repeated hospitalizations, ECOG-PS ≥ 2, progression after several treatment lines); (b) provide structured, easily visible templates for documenting ACP and goals-of-care; (c) trigger palliative care referrals when documentation indicates limits on life-prolonging treatment; (d) automatically generate LOI-15 and LOI-30 indicators as part of institutional quality dashboards [4,5,6,7,8,9,19,27,28]. More sophisticated applications, such as machine-learning models that flag patients at high risk of receiving aggressive EoL care misaligned with their preferences, remain experimental but could, in the future, complement clinician judgment—provided they are grounded in robust, ethically collected data and embedded in workflows that prioritize dialog over automation.

### 4.3. Strengths and Limitations

This study has several strengths. It uses real-world EHR data from a middle-income country, providing insights into EoL care patterns beyond high-income settings where most evidence originates [4,5,6,7,8,9,10,23]. It operationalizes LOI-15 and LOI-30 using concrete treatment data and applies Poisson regression to estimate relative risks, which are more intuitive than odds ratios for clinicians and policy-makers. The focus on ACP documentation and its visibility in a digital environment connects empirical findings directly with the theme of the digital era and with ongoing international efforts to embed ACP into routine care through electronic systems [19,23,24]. Data abstraction followed a standardized form with double review of ambiguous cases, which reduces (although does not eliminate) the risk of misclassification.

Nevertheless, limitations must be acknowledged. The sample size was modest, especially for LOI-15, resulting in wide confidence intervals and an increased risk of type II error. Data abstraction, though systematic, may still have been subject to information bias and under-documentation. The chart-abstraction duration was not prospectively recorded and could not be reliably reconstructed. Time from diagnosis to death was considered, but diagnosis dates were not consistently available as structured EHR data, so it was not included. As in all retrospective chart reviews, ACP discussions, symptom burden and contextual details (e.g., family pressure, patient preferences expressed verbally but not recorded) were not fully captured [12,13,14,15,16,17,18,19]. Tumor types and treatment pathways were heterogeneous, and we could not adjust for all nuances without overfitting. Detailed tumor histology, staging and line-of-therapy information were inconsistently recorded in structured fields, and primary site information was therefore handled in broad categories; as a result, we did not adjust for tumor type in multivariable models. Our composite LOI outcome combines potentially futile and appropriate symptom-directed interventions, limiting inferences at the individual level. Finally, the study was conducted in only two centers; generalizability to other regions or institutions with different levels of palliative care integration, digital infrastructure or resource constraints may be limited.

### 4.4. Implications for Practice, Policy and Digital Ethics

Despite these limitations, the findings have practical implications. Clinicians should be aware that younger and more educated patients may be at risk of receiving more intensive treatment near death, potentially driven by a combination of expectations, social capital and therapeutic inertia [4,5,6,7,8,9,10,23]. Awareness of this pattern can prompt more deliberate, values-based conversations about prognosis and treatment goals, particularly when laboratory parameters and functional status suggest limited benefit from further disease-directed therapy.

At the system level, health services that already rely on digital records can incorporate ACP visibility and LOI indicators into quality improvement initiatives. Monitoring LOI-30 over time, stratified by age, education, tumor type and ACP status, may help identify areas where aggressive EoL care is inconsistent with patient preferences or evidence-based guidelines [4,5,6,7,8,9,19]. Policymakers in middle-income countries can consider incentivizing ACP documentation and palliative care integration as part of broader digital health strategies, while explicitly addressing issues of access, literacy and equity [17,19,27,28].

From a bioethical perspective, our results reinforce that digital tools are not morally neutral. Their design and deployment can either promote or undermine core values in EoL care. Tools that make ACP visible, invite reflection on proportionality and highlight LOI patterns can support beneficence and non-maleficence by reducing futile or burdensome interventions. Tools that are co-designed with patients and families, attentive to language and literacy, can promote autonomy and justice. In contrast, tools that prioritize throughput, billing or defensive documentation over patient narratives risk aggravating the very problems they aim to solve. Any digital strategy for improving EoL care should therefore be grounded not only in technical feasibility but also in explicit bioethical analysis.

## 5. Conclusions

Using EHR-based digital records in a Brazilian cohort of patients with metastatic solid tumors, this study provides new evidence on the relationship between ACP visibility and LOI near death, while characterizing clinical and sociodemographic factors associated with high-intensity care in a middle-income setting. Lower platelet counts—a simple marker of hematologic reserve—were paradoxically associated with a higher likelihood of LOI, suggesting potential therapeutic inertia in frail patients. ACP documentation in the EHR, although rare, was associated with the complete absence of LOI-30.

These findings underscore the importance of integrating ACP into routine oncology care and of using existing digital tools—such as EHR-based prompts, structured ACP templates and LOI indicators—to improve EoL decision-making [12,13,14,15,16,17,18,19,27,28]. In the digital era, the challenge is less about inventing new technologies and more about using current systems to make patient values and goals-of-care visible at the bedside, thereby reducing the risk of potentially futile late-stage cancer treatments.

Future work should focus on developing and evaluating digital tools explicitly centered on the needs and values of patients with advanced cancer and their families, particularly in middle-income countries. Such tools should support the identification of high-risk patients, facilitate ACP discussions, and help monitor quality indicators like LOI-15 and LOI-30, while being carefully adapted to local levels of literacy, connectivity and resource availability [17,19,27,28]. Crucially, digital tools must be designed to support, but never replace, direct human contact: face-to-face conversations, empathic listening and shared deliberation remain irreplaceable for truly patient-centered and ethically robust end-of-life care.

## Figures and Tables

**Table 1:** Sociodemographic and clinical characteristics (*N* = 79).

Variable	Value
Sociodemographic characteristics	
Age at death, years	65.9 ± 14.7
Sex–Male	42 (53.2%)
Sex–Female	37 (46.8%)
Elementary education	35 (44.3%)
High school	36 (45.6%)
Higher education	8 (10.1%)
Cancer type	
Breast cancer	15 (19.0%)
Lung cancer	13 (16.5%)
Other solid tumors	51 (64.6%)
Comorbidities	
Hypertension	48 (60.8%)
Diabetes mellitus	14 (17.7%)
Disease extent	
Oligometastatic disease	11 (13.9%)
Extensive disease	68 (86.1%)
Performance status	
ECOG 0–1 *	15 (20.6%)

* Percentage calculated among patients with available ECOG-PS data (*N* = 73).

**Table 2:** Late oncologic interventions near death (*N* = 79).

Outcome/Intervention	*N* (%)
Any LOI ≤ 30 days	17 (21.5%)
Any LOI ≤ 15 days	7 (8.9%)
Systemic therapy (LOI-30)	10 (58.8%) *
Radiotherapy (LOI-30)	5 (29.4%) *
Oncologic surgery (LOI-30)	2 (11.8%) *

* Percentages calculated among the 17 patients who received at least one LOI-30. Some patients received more than one modality.

**Table 3:** Multivariable Poisson regression for late oncologic interventions (*N* = 79).

Outcome	Predictor	RR	95% CI	*p*-Value
LOI ≤ 15 days	Age < 60 years	8.19	0.73–91.6	0.088
LOI ≤ 15 days	Higher education	8.74	2.57–29.7	<0.001
LOI ≤ 15 days	Any comorbidity	0.92	0.28–3.04	0.894
LOI ≤ 15 days	Oligometastatic disease	1.65	0.38–7.06	0.500
LOI ≤ 15 days	Platelet count (per 10,000/µL)	0.94	0.89–0.96	0.010
LOI ≤ 30 days	Age < 60 years	3.76	1.50–9.44	0.005
LOI ≤ 30 days	Higher education	2.07	1.07–3.99	0.030
LOI ≤ 30 days	Any comorbidity	0.69	0.35–1.39	0.305
LOI ≤ 30 days	Hypertension	0.59	0.28–1.24	0.162
LOI ≤ 30 days	Oligometastatic disease	1.38	0.76–2.50	0.291
LOI ≤ 30 days	Platelet count (per 10,000/µL)	0.96	0.92–0.99	0.020
LOI ≤ 30 days	Leukocyte count (per 1000 cells/mm^3^)	0.93	0.79–1.08	0.337

## Data Availability

The data presented in this study are available on request from the corresponding author. Due to Brazilian data protection laws and the small size of the cohort, the dataset is not publicly available. De-identified data may be made available upon reasonable request to the corresponding author, subject to institutional approvals and additional ethical review, where applicable.
